# Optimizing Siglec-8-Directed Immunotherapy for Eosinophilic and Mast Cell Disorders

**DOI:** 10.3390/cancers16203476

**Published:** 2024-10-14

**Authors:** Sheryl Y. T. Lim, Jenny Huo, George S. Laszlo, Frances M. Cole, Allie R. Kehret, Junyang Li, Margaret C. Lunn-Halbert, Jasmyn L. Persicke, Peter B. Rupert, Roland K. Strong, Roland B. Walter

**Affiliations:** 1 Translational Science and Therapeutics Division, Fred Hutchinson Cancer Center, Seattle, WA 98109, USA; ylim2@fredhutch.org (S.Y.T.L.); glaszlo@fredhutch.org (G.S.L.); jli3@fredhutch.org (J.L.); mlunn1@uw.edu (M.C.L.-H.);; 2 Basic Sciences Division, Fred Hutchinson Cancer Center, Seattle, WA 98109, USA; prupert@fredhutch.org (P.B.R.); rstrong@fredhutch.org (R.K.S.); 3 Department of Medicine, Division of Hematology and Oncology, University of Washington, Seattle, WA 98195, USA; 4 Department of Laboratory Medicine and Pathology, University of Washington, Seattle, WA 98195, USA

**Keywords:** Siglec-8, immunotherapy, eosinophils, mast cells, chimeric antigen receptor

## Abstract

**Simple Summary:**

Siglec-8 has been identified as a promising target to treat eosinophilic and mast cell disorders. However, there are currently few Siglec-8 antibodies available, and therapeutic efforts have so far primarily focused on unconjugated antibodies, which may be insufficiently effective in many patients. To address these limitations, we raised a diverse panel of fully human Siglec-8 antibodies as the basis for novel therapeutics. They were all efficiently internalized, suggesting their potential to deliver cytotoxic payloads. T cell-engaging bispecific antibodies and chimeric antigen receptor (CAR)-modified natural killer (NK) cells built from these Siglec-8 antibodies were highly potent against Siglec-8-positive cells even in cases of very low target antigen abundance. Importantly, mechanistic studies with target cells expressing either full-length Siglec-8 or an artificial smaller Siglec-8 variant demonstrated that T cell-engaging bispecific antibodies and CAR-modified NK cells were substantially more effective if they bound Siglec-8 closer to the cell membrane, indicating targeting membrane-proximal epitopes enhances effector functions of Siglec-8 antibody-based therapeutics. Indeed, tool therapeutics that bind one of the membrane-proximal C2-set domains of Siglec-8 were very effective against Siglec-8-positive target cells. Together, these data demonstrate Siglec-8-directed immunotherapies can be highly potent, supporting their further development for eosinophilic and mast cell disorders.

**Abstract:**

**Background/Objective:** Current treatments for eosinophilic and mast cell disorders are often ineffective. One promising target to improve outcomes is sialic acid-binding immunoglobulin-like lectin-8 (Siglec-8). As limitations, there are few Siglec-8 monoclonal antibodies (mAbs) available to date, and Siglec-8-directed treatments have so far primarily focused on unconjugated mAbs, which may be inadequate, especially against mast cells. **Methods:** Here, we used transgenic mice to raise a diverse panel of fully human mAbs that either recognize the V-set domain, membrane-distal C2-set domain, or membrane-proximal C2-set domain of full-length Siglec-8 as a basis for novel therapeutics. **Results:** All mAbs were efficiently internalized into Siglec-8-expressing cells, suggesting their potential to deliver cytotoxic payloads. Tool T cell-engaging bispecific antibodies (BiAbs) and chimeric antigen receptor (CAR)-modified natural killer (NK) cells using single-chain variable fragments from Siglec-8 mAbs showed highly potent cytolytic activity against Siglec-8-positive cells even in cases of very low target antigen abundance, whereas they elicited no cytolytic activity against Siglec-8-negative target cells. Siglec-8^V-set^-directed T cell-engaging BiAbs and Siglec-8^V-set^-directed CAR-modified NK cells induced substantially greater cytotoxicity against cells expressing an artificial smaller Siglec-8 variant containing only the V-set domain than cells expressing full-length Siglec-8, consistent with the notion that targeting membrane-proximal epitopes enhances effector functions of Siglec-8 antibody-based therapeutics. Indeed, unconjugated Siglec-8^C2-set^ mAbs, Siglec-8^C2-set^-directed T cell-engaging BiAbs, and Siglec-8^C2-set^-directed CAR-modified NK cells showed high antigen-specific cytolytic activity against Siglec-8-positive human cell lines and primary patient eosinophils. **Conclusions:** Together, these data demonstrate Siglec-8-directed immunotherapies can be highly potent, supporting their further development for eosinophilic and mast cell disorders.

## 1. Introduction

Eosinophils and/or mast cells play an important role in many diseases [1,2,3,4,5]. Current treatments include monoclonal antibodies (mAbs) against interleukin-5 (e.g., mepolizumab, reslizumab) or the interleukin-5 receptor (e.g., benralizumab) to target eosinophils or mAbs that bind free IgE (e.g., omalizumab) to block mast cells. However, these and other existing therapeutics are often insufficiently effective, and better treatments are needed. One promising target for this purpose is sialic acid-binding immunoglobulin-like lectin-8 (Siglec-8; also known as SAF2) [1,2,3,4,5,6,7].

In a randomized trial, the humanized non-fucosylated IgG1 Siglec-8 mAb, lirentelimab (AK002), reduced eosinophils and alleviated symptoms in patients with eosinophilic gastritis and duodenitis [8], validating Siglec-8 as a drug target. Results from small, non-controlled trials also suggest benefit with lirentelimab in other eosinophilic/mast cell diseases [9,10,11]. However, there are currently few Siglec-8 mAbs available, and how Siglec-8 is optimally targeted is unknown. In vitro, unconjugated Siglec-8 mAbs like lirentelimab deplete eosinophils and inhibit mast cell function, but they do not induce apoptosis of mast cells [4,12,13,14], indicating they may be inadequate against some Siglec-8-positive disorders. This is reminiscent of the clinical experience with Siglec-3 (CD33), where unconjugated mAbs have been largely ineffective. More potent modalities, e.g., T cell- or natural killer (NK) cell-directed bispecific antibodies (BiAbs) or chimeric antigen receptor (CAR)-modified immune effector cells, may thus be required for successful Siglec-8-targeted therapy. Our data with CD33-targeted therapeutics suggest the efficacy of Siglec-directed therapy is also affected by the location of the epitope to which they bind. Specifically, we found membrane-proximal binding enhances effector functions of CD3-directed BiAbs and CAR-modified T cells [15,16]. To optimize Siglec-8-directed therapy, we used transgenic mice to raise human Siglec-8 mAbs. We then examined the role of membrane-proximal targeting as a way of enhancing the cytotoxicity of Siglec-8-targeted therapeutics and generated tool BiAbs engaging T cells and CAR-modified NK cells as novel treatments for Siglec-8-positive disorders.

## 2. Materials and Methods

### 2.1. Expression Vectors Encoding Siglec-8 Variants

Full-length Siglec-8 (Siglec-8^FL^) with/without an N-terminal 6-histidine tag was generated using the endogenous signal peptide (amino acids [aa] 1–16) and the human Siglec-8 coding region, including the extracellular domain (ECD, aa 17–363), transmembrane domain (aa 364–384), and intracellular domain (aa 385–499). A truncated Siglec-8 lacking exon-2 (Siglec-8^ΔE2^) was generated with the signal peptide, the V-set domain (aa 17–151), and aspartic acid at position 152 (site of exon 1/3 junction produced by exon 2-skipping) followed by the membrane-proximal C2-set domain and all remaining Siglec-8 amino acids (aa 246–499). Another truncated Siglec-8 containing only the V-set domain in the ECD (Siglec-8^Vset^) was generated with the signal peptide, the V-set domain (aa 17–152), the juxtamembrane region (aa 352–363), and all remaining Siglec-8 amino acids (aa 364–499). For immunizations, we used Siglec-8^FL^ and/or chimeric proteins consisting of the V-set domain of murine CD33 (aa 1–233) fused with the membrane-proximal C2-set domain, transmembrane and intracellular domain of human Siglec-8 (aa 246–499), or the V-set portion of murine Siglec-F (aa 1–227) fused to the membrane-proximal C2-set domain of human Siglec-8 (aa 246–499). cDNAs, synthesized as gBlocks (Integrated DNA Technologies; Coralville, IA, USA), were cloned into pRRLsin.cPPT.MSCV lentivirus constructs containing an IRES-Enhanced Green Fluorescent Protein (EGFP) cassette. All constructs were sequence-verified before use.

### 2.2. Genetic Deletion of Siglec-8

For CRISPR/Cas9 editing, purified Cas9 protein (TrueCut Cas9 V2; Thermo Fisher Scientific; Waltham, MA, USA) complexed with a pool of 3 synthetic guide RNAs (sgRNA) targeting Siglec-8 (5′-CUGGGGGUAGGAGAAGGAGC-3′, 5′-CUUGCUGCAAGUGCAGGAGC-3′, and 5′-CCCCUUUGUCCCCCAGAGCA-3′) was electroporated into Siglec-8-positive target cells [15,16,17,18]. The resulting Siglec-8-negative single cells were FACS isolated, expanded, and their genomic DNA amplified/analyzed to confirm frame-shift mutation/gene disruption at all Siglec-8 loci.

### 2.3. Parental and Engineered Human Mast Cells, Human Acute Leukemia Cells, and NK Cell Lines

LUVA cells (Kerafast; Boston, MA, USA) were maintained in StemPro-34 SFM (Thermo Fisher Scientific) and 2 mM L-glutamine. The human mast cell line HMC-1.2 (Sigma-Aldrich; St. Louis, MO, USA) was cultured in IMDM with 25 mM HEPES, 3.024 g/L sodium bicarbonate, 10% fetal bovine serum (FBS) (Hyclone; Thermo Fisher Scientific), 1% L-glutamine, and 0.1% β-mercaptoethanol (all from Gibco, Thermo Fisher Scientific). KHYG-1 cells (German Collection of Microorganisms and Cell Cultures [DSMZ]; Braunschweig, Germany) were cultured in RPMI-1640 with 10% FBS and 100 U/mL of recombinant IL-2 (Peprotech; Cranbury, NJ, USA). Human myeloid HL-60, K562, ML-1, and TF-1 cells as well as human lymphoid RS4;11 cells were cultured as described [15,18]. Human myeloid EOL-1 cells were cultured in RPMI-1640 with 10% FBS. Lentivirally transduced sublines of various cell lines were generated at multiplicities of infection of 1–25; EGFP-positive cells were isolated by FACS and re-cultured for further analysis/use. All cell lines were grown with penicillin/streptomycin, routinely tested for mycoplasma contamination (MycoAlert Mycoplasma Detection Kit; Lonza; Basel, Switzerland), and authenticated using standard STR CODIS typing.

### 2.4. Primary Human NK Cells

Non-mobilized peripheral blood cells were collected via leukapheresis from healthy adult volunteers after informed consent was obtained by an institutional core facility under a research protocol approved by the Fred Hutchinson Cancer Center (FHCC) Institutional Review Board (IRB). Subsequently, primary human NK cells were isolated by CD56-positive selection using cliniMACS microbeads (Militenyi Biotec; Bergisch Gladbach, Germany) and then rested in RPMI-1640 with 10% heat-inactivated pooled human serum (Bloodworks Northwest; Seattle, WA, USA), 3.5 mM glutamine, 44 µM β-mercaptoethanol, 100 µg/mL penicillin/streptomycin, and supplemented with 500 U/mL of recombinant IL-2 (Peprotech) at 37 °C in 5% CO_2_ overnight before freezing. Frozen purified NK cells, provided for research purposes in de-identified fashion, were thawed 1 day before use.

### 2.5. Primary Human Eosinophils

Peripheral blood specimens from two patients with eosinophilia (Supplementary Table S1) were obtained under a research protocol approved by the FHCC IRB (FH #1690). Under this protocol, both patients provided written informed consent for collection and research use of samples. Eosinophils were isolated with the Eosinophil Isolation Kit (Miltenyi Biotec) with purity of enriched eosinophils (CCR3+, Siglec-8+) exceeding 90%, as evaluated by a BD FACSymphony A5 flow cytometry.

### 2.6. Generation of Human Siglec-8 mAbs

Alloy ATX-GK and ATX-GL mice (Alloy Therapeutics; Waltham, MA, USA) were injected with combinations of mouse 3T3 cells expressing different Siglec-8 immunogens. Hybridoma screening was performed flow cytometrically using K562 sublines overexpressing either Siglec-8^FL^ or chimeric mouse/human versions of Siglec-8 expressing only the juxtamembrane portion of the ECD of Siglec-8. Hybridomas with reactivity against Siglec-8^FL^ and/or reactivity against the juxtamembrane portion of Siglec-8′s ECD were subcloned and expanded for further use.

### 2.7. Expression and Purification of Recombinant Human Siglec-8 mAbs and Negative Control mAb

Additionally, 5′ rapid amplification of cDNA ends (RACE) cloning of antibody sequences was performed as per company instructions (Alloy Therapeutics). Protein sequences were reverse-translated using human codons for cloning, cDNA was synthesized as gBlocks for cloning into pcDNA3.4 vectors, sequences were verified, and heavy chain and light chain pairs were transiently transfected into Expi293 cells (Thermo Fisher Scientific) with human IgG1 and IgG4 (S228P) frameworks. mAbs were then purified using protein A affinity chromatography on an AKTA pure system with MAbSelectSuRe columns (Cytiva; Marlborough, MA, USA), quantified, and analyzed by SDS-PAGE. A negative control mAb, 13R4 (recognizing *E. coli* beta-galactosidase), was generated and purified in a similar fashion using published sequences (European patent EP2134841B1; SEQ ID NO 33). No changes were made to antibody glycosylation. Lirentelimab was obtained from MedChemExpress (Monmouth Junction, NJ, USA).

### 2.8. Quantification of Siglec-8 Expression

Expression of Siglec-8 was quantified by flow cytometry on a BD FACSCelesta cytometer using a phycoerythrin (PE) directly labeled mAb (7C9; BD Biosciences; Franklin Lakes, NJ, USA). QuantiBRITE PE beads were used to standardize quantitation of antibody molecules bound/cell (BD Biosciences). To identify non-viable cells, samples were stained with 4′,6-diamidino-2-phenylindole (DAPI), and DAPI-negative cells were analyzed using FlowJo version 10 (BD Biosciences).

### 2.9. Construction, Expression, and Purification of Siglec-8/CD3- and CD19/CD3-Directed BiAbs

T cell-engaging Siglec-8-directed (i.e., Siglec-8/CD3) BiAbs were constructed in the single-chain variable fragment (scFv)-scFv format using published sequences (United States patent US 10,933,132 B2; SEQ ID NO 227) [19], with the CD33-binding scFv portion replaced by Siglec-8-binding scFvs. A CD19/CD3 BiAb was generated in the scFv-scFv format with published sequences available in United States patent US 8,007,796 B2 (SEQ ID NO 2) [19]. Protein sequences were reverse-translated using human codons, cDNA was synthesized as gBlock for cloning into pcDNA3.4 vectors, and sequences were verified. Proteins were expressed transiently as described above and purified using nickel affinity chromatography on an AKTA pure system with HisTrap columns (Cytiva).

### 2.10. Quantification of Siglec-8 Internalization

To quantify Siglec-8 internalization, cells were stained with 2 µg/mL unlabeled Siglec-8 IgG4 mAb in staining buffer incubated in ice water for 20 min. After washing in phosphate-buffered saline (PBS), cells were split in aliquots and either left in ice water or incubated at 37 °C for various periods of time. Samples were then stained with PE-conjugated goat anti-human IgG Fc secondary mAb (SouthernBiotech; Birmingham, AL, USA) to identify remaining mAbs on the cell surface, and fluorescence was quantified flow cytometrically.

### 2.11. CAR-NK Cell Generation

CAR-modified KHYG-1 cells were generated through lentiviral transduction. Briefly, Siglec-8 scFvs were generated as gBlocks, cloned into the lentiviral vector pSLCAR-CD19-BBz (plasmid # 135992 [Addgene.org], containing a 60 aa hinge domain spacer, CD28 transmembrane domain, CD3zeta and 4-1BB intracellular signaling domain, and a P2A-EGFP reporter transduction marker) to generate pSLCAR-Siglec-8-BBz constructs, and sequence-verified by Sanger sequencing. Lentivirally transduced cell sublines were identified and isolated as described above.

### 2.12. Quantification of Antibody-Dependent Cellular Cytotoxicity (ADCC) and BiAb- and CAR-NK Cell-Induced Cytotoxicity

BiAb-induced cytotoxicity was determined flow cytometrically, using human T cells enriched from unstimulated peripheral blood mononuclear cells collected from healthy volunteers [15,19,20,21,22,23,24,25,26]. ADCC was determined flow cytometrically using healthy donor NK cells. Either the effector cells or target cells were labeled with CellVue Burgundy Labeling Kit (ThermoFisher Scientific) to differentiate cell types. A BD FACSCelesta cytometer was used for experiments involving cell lines, in which cytotoxicity was quantified either as a change in the percentage of dead (i.e., DAPI-positive) cells or as drug-specific cytotoxicity [21,26]. For experiments with primary human eosinophils, unlabeled eosinophils were co-cultured with healthy donor NK cells or T cells or CAR-NK cells with or without increasing concentrations of the respective mAbs or BiAbs for 16–18 h before staining with an AF647-labeled CCR3 mAb (5E8; BioLegend; San Diego, CA, USA), PE-labeled Siglec-8 mAb (7C9; BioLegend) and APC-Cy7-labeled CD16 mAb (3G8; BD Biosciences), followed by DAPI. A BD FACSymphony A5 cytometer was used for primary eosinophil cytotoxicity experiments, in which cytotoxicity was quantified as a change in the number of viable eosinophils: %Cytotoxicity = 100 × (1 − live eosinophils treated/live eosinophils control).

### 2.13. Statistical Analysis

Comparisons of Siglec-8 expression levels and drug-induced cytotoxicity were performed with Prism 10 (GraphPad; San Diego, CA, USA) using repeat-measure two-way ANOVA with multiple comparison testing.

## 3. Results

### 3.1. Production and Characteristics of New Human Siglec-8 mAbs

**Figure 1:**
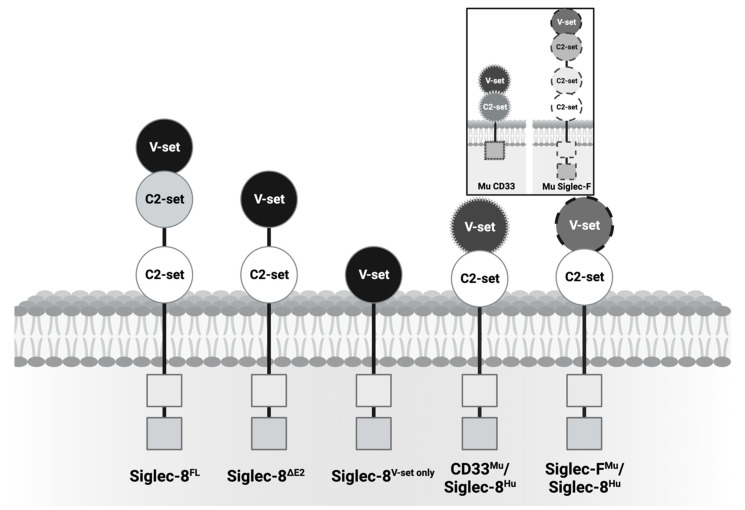
Schematic of full-length Siglec-8 (Siglec-8^FL^) and artificial Siglec-8 proteins with deletion of the membrane-distal C2-set domain or deletion of both the membrane-proximal and membrane-distal C2-set domain, as well as chimeric Siglec-8 molecules in which the membrane-proximal C2-set domain of human (Hu) Siglec-8 is fused to the V-set domain of either murine (Mu) CD33 or Mu Siglec-F. Figure was generated with BioRender (https://biorender.com).

**Figure 2:**
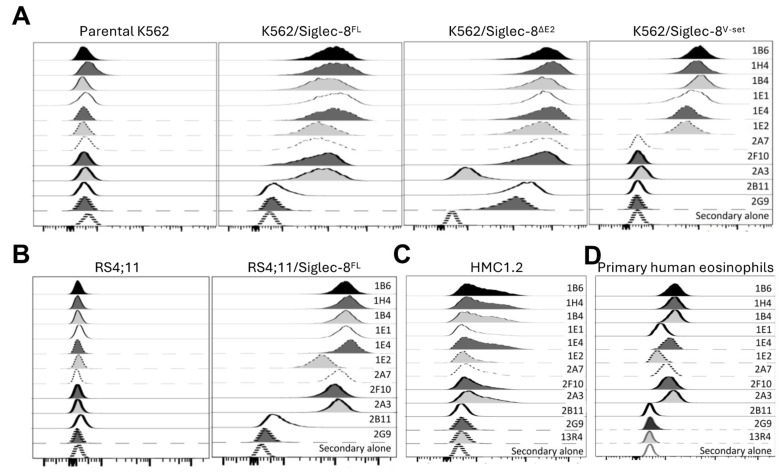
Specific binding of Siglec-8 mAbs. Siglec-8 mAbs were tested against (**A**) parental human myeloid K562 cells (endogenously lacking Siglec-8 expression) and K562 sublines overexpressing Siglec-8^FL^, Siglec-8^ΔE2^, or Siglec-8^V-set^, (**B**) parental human lymphoid RS4;11 cells (endogenously lacking Siglec-8 expression) and a subline overexpressing Siglec-8^FL^, (**C**) parental human HMC-1.2 enriched for expression of endogenous Siglec-8, and (**D**) primary human eosinophils isolated from a patient (representative sample of n = 2). In all experiments, a negative control without primary mAbs was included; in experiments with HMC-1.2 and primary human eosinophils, a second negative control with a non-binding primary mAb (13R4) was additionally included.

We immunized transgenic mice with human Siglec-8^FL^ or a chimeric human/mouse protein, which consisted of the membrane-proximal C2-set domain of human Siglec-8 fused with either the V-set domain of murine CD33 or the V-set domain of murine Siglec-F (Figure 1). Hybridomas of interest were selected based on binding to K562 cells overexpressing either human Siglec-8^FL^ or truncated Siglec-8 proteins, and mAb variable domain regions were sequenced. Eleven mAbs recognizing human Siglec-8 and/or variants thereof were identified and produced as recombinant proteins for further characterization. Nine of these 11 mAbs efficiently recognized Siglec-8^FL^ (Figure 2A): six of the mAbs bound the V-set domain (1B6, 1H4, 1B4, 1E1, 1E4, and 1E2), one mAb bound the membrane-distal C2-set domain (2A3), and two mAbs bound the membrane-proximal C2-set domain (2A7 and 2F10). These nine mAbs recognized Siglec-8^FL^ expressed on genetically engineered human lymphoid RS4;11 cells (Figure 2B) as well as Siglec-8 endogenously expressed on both HMC-1.2 cells and primary human eosinophils isolated from patients with eosinophilia (Figure 2C,D). In contrast, two of the 11 mAbs (2B11 and 2G9) only efficiently recognized a truncated Siglec-8 protein consisting of the V-set domain and the membrane-proximal C2-set domain but not Siglec-8^FL^ (Figure 2A) or endogenous Siglec-8 (Figure 2C,D), indicating binding to a truncation-specific epitope.

### 3.2. Internalization of Siglec-8 mAbs

**Figure 3:**
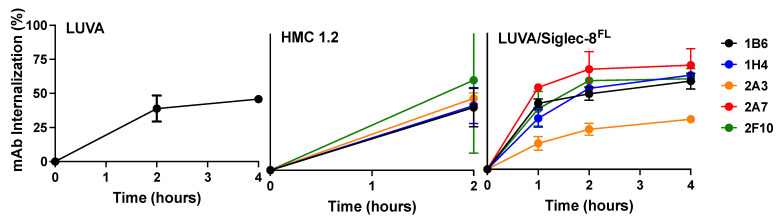
Siglec-8 mAb internalization. Internalization of Siglec-8 IgG4 mAbs (1B6, 1H4, 2A3, 2A7, 2F10) in parental LUVA cells (**left panel**), parental human HMC-1.2 enriched for expression of endogenous Siglec-8 (**middle panel**), and LUVA cells overexpressing Siglec-8^FL^ (**right panel**). Internalization was calculated as 1 − ([MFI_time X_ − MFI_isotype control time X_]/[MFI_time 0_ − MFI_isotype control time 0_]). Shown are mean ± SEM of 2–3 independent experiments.

Because Siglec-8 has endocytic properties [27], we performed flow cytometry-based internalization assays to characterize our Siglec-8 mAbs as carriers for cytotoxic payloads. For these experiments, we used parental LUVA cells, which endogenously express very low levels of Siglec-8, a subline of LUVA cells in which we overexpressed Siglec-8^FL^, and, as a negative control, a subline of LUVA cells in which we deleted the Siglec-8 gene loci via CRISPR/Cas9. We also assessed internalization of Siglec-8 mAbs in HMC-1.2 cells. Since this cell line only expressed Siglec-8 in a subset of cells, we used FACS to enrich HMC-1.2 cells for Siglec-8 expression for our assays (Figure 2C). All Siglec-8 mAbs we tested were internalized time dependently by parental and Siglec-8-overexpressing LUVA cells as well as HMC-1.2 cells (Figure 3). In contrast, no internalization was observed in Siglec-8-deleted LUVA cells, demonstrating that the Siglec-8 mAb uptake seen in LUVA cells was dependent on Siglec-8 expression.

### 3.3. Efficacy of Siglec-8-Directed Immunotherapies Engaging T or NK Cells

**Figure 4:**
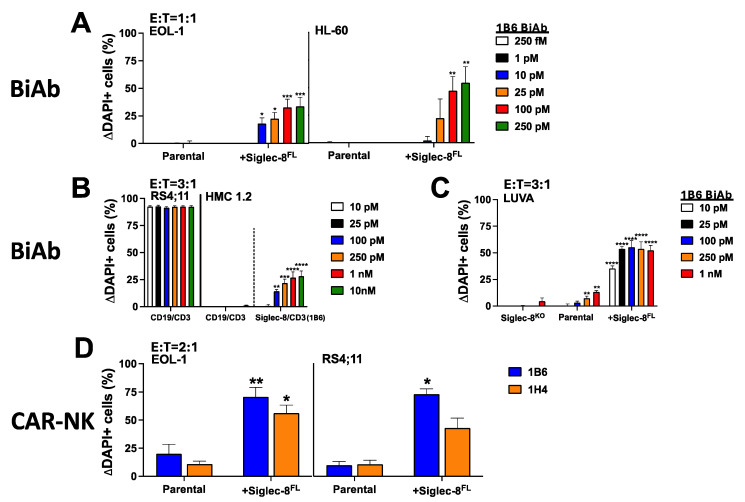
Cytolytic activity of Siglec-8^V-set^ therapies engaging T or NK cells. (**A**) Parental EOL-1 and HL-60 cells and corresponding sublines overexpressing Siglec-8^FL^ were incubated with healthy donor T cells at an E:T cell ratio of 1:1 with increasing concentrations of a Siglec-8^V-set^/CD3 BiAb (1B6). (**B**) Parental CD19+ RS4;11 cells and HMC-1.2 cells enriched for expression of endogenous Siglec-8 were incubated with healthy donor T cells at an E:T cell ratio of 3:1 with increasing concentrations of a CD19/CD3 or a Siglec-8^V-set^/CD3 BiAb (1B6). (**C**) Siglec-8 gene-edited LUVA cells (Siglec-8^KO^), parental LUVA cells, and LUVA cells overexpressing Siglec-8^FL^ were incubated with healthy donor T cells at an E:T cell ratio of 3:1 with increasing concentrations of a Siglec-8^V-set^/CD3 BiAb (1B6). (**D**) Parental EOL-1 and RS4;11 cells and corresponding sublines overexpressing Siglec-8^FL^ were incubated at an E:T cell ratio of 2:1 with KHYG-1 cells expressing a Siglec-8^V-set^-directed CAR (CAR-NK) (1B6 or 1H4). For all experiments, non-viable target cells were enumerated after 48 h via flow cytometry. Change in dead cells with treatment compared to cells without treatment is shown (mean + SEM from 3 separate experiments). * *p* < 0.05; ** *p* < 0.01; *** *p* < 0.001; **** *p* < 0.0001.

So far, Siglec-8-directed therapy has been largely pursued with unconjugated mAbs. However, since unconjugated mAbs are often ineffective, perhaps especially at lower target antigen levels, we assessed the value of Siglec-8-directed therapies engaging T or NK cells. For this purpose, we generated T cell-engaging Siglec-8/CD3 BiAbs (scFv-scFv format) and Siglec-8 CAR-NK cells as tool agents, using binding sequences from Siglec-8^V-set^ mAbs. As shown in Figure 4A,C, very low concentrations of a Siglec-8/CD3 BiAb (1B6) induced dose-dependent toxicity against EOL-1 and HL-60 as well as LUVA cells transduced with human Siglec-8^FL^. This BiAb also elicited Siglec-8-specific cytolytic activity against HMC-1.2 cells that were enriched for Siglec-8 expression as well as parental LUVA cells, both cell types which display very minimal numbers of Siglec-8 molecules (Figure 4B,C). The observed activity of the CD19/CD3 BiAb against CD19+ RS4;11 cells but not CD19- HMC-1.2 cells strongly suggested that the cytotoxicity of the Siglec-8/CD3 BiAb against HMC-1.2 cells was not due to non-specific killing from T cells engaged via CD3 alone (Figure 4B). Likewise, KHYG-1 cells expressing a Siglec-8^V-set^-directed CAR integrating scFv sequences from 1B6 or 1H4 elicited significant cytotoxicity against Siglec-8^FL^-transduced EOL-1 and RS4;11 cells (Figure 4D). Together, these studies demonstrated Siglec-8-directed therapies engaging T or NK cells can be highly effective against Siglec-8-expressing cells, even when displaying very low amounts of the target antigen.

### 3.4. Binding Distance from Cell Membrane Correlates with Efficacy of Siglec-8-Directed Therapies Engaging T or NK Cells

**Figure 5:**
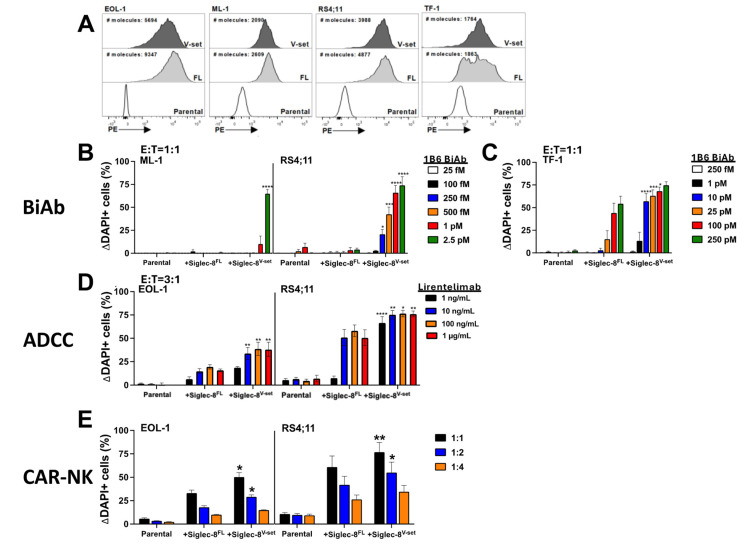
Membrane proximity of the target epitope modulates the efficacy of Siglec-8^V-set^-directed BiAbs, ADCC, and CAR-NK cells. (**A**) Parental EOL-1, ML-1, RS4;11, and TF-1 cells were used to generate sublines expressing either Siglec-8^FL^ or a Siglec-8 variant containing only the V-set domain (Siglec-8^V-set^). Relative expression of the target proteins was then flow cytometrically quantified. (**B**) Parental ML-1, RS4;11, and (**C**) TF-1 cells and corresponding sublines overexpressing similar levels of Siglec-8^FL^ or Siglec-8^V-set^ were incubated with healthy donor T cells at an E:T cell ratio of 1:1 with increasing concentrations of a Siglec-8^V-set^/CD3 BiAb (1B6). (**D**) Parental EOL-1 and RS4;11 cells and corresponding sublines overexpressing similar levels of Siglec-8^FL^ or Siglec-8^V-set^ were incubated with healthy donor NK cells at an E:T cell ratio of 3:1 with increasing concentrations of the Siglec-8^V-set^ mAb, lirentelimab. (**E**) Cell lines as in (**D**) were incubated at different E:T cell ratios with KHYG-1 cells expressing a Siglec-8^V-set^-directed CAR (1B6) (CAR-NK). For all experiments, non-viable target cells were enumerated after 48 h via flow cytometry. Change in dead cells with treatment compared to cells without treatment is shown (mean + SEM from at least 3 separate experiments). * *p* < 0.05; ** *p* < 0.01; *** *p* < 0.001; **** *p* < 0.0001.

In previous studies aimed at optimizing therapies targeting Siglec-3 (CD33), we found that membrane-proximal binding enhances CD3-directed BiAbs and CAR-modified T cells [15,16]. Herein, we employed an experimental approach similar to the one we used in those studies to examine the relationship between the efficacy of T cell- and NK cell-engaging therapies and the distance between the target domain on Siglec-8 and the cell membrane [15,16]. Specifically, using human acute myeloid leukemia (AML) and acute lymphoblastic leukemia (ALL) cell lines, we engineered cell line pairs overexpressing comparable levels of Siglec-8^FL^ or an artificial variant of Siglec-8 with only the V-set domain (Siglec-8^V-set^) to juxtapose the V-set domain to the cell membrane (Figure 1) as target cells for short-term in vitro cytotoxicity assays (Figure 5A). The ML-1, RS4;11, and TF-1 cell lines express between 2000 and 10,000 Siglec-8 molecules per cell (Figure 5A). For comparison, there are ~18,000–22,000 Siglec-8 molecules/cell on eosinophils and ~500 Siglec-8 molecules/cell on basophils [6]. The cell line pairs were subjected to such assays with various doses of the Siglec-8^V-set^/CD3 BiAb and healthy human T cells as effectors. As shown in Figure 5B,C, Siglec-8^V-set^/CD3 BiAbs exerted greater cytotoxicity against AML and ALL cells expressing Siglec-8^V-set^ than cells expressing Siglec-8^FL^ despite similar or higher expression levels of Siglec-8^FL^. To examine the relationship between ADCC and the Siglec-8 mAb binding distance from the cell membrane, EOL-1 and RS4;11 cell line pairs were then subjected to similar assays with increasing concentrations of Siglec-8^V-set^ mAbs (lirentelimab, 1H4) and healthy human NK cells as effectors. Both lirentelimab (Figure 5D) and 1H4 (Supplementary Figure S1) exerted greater ADCC against cells expressing Siglec-8^V-set^ than cells expressing Siglec-8^FL^ despite similar or higher abundance of Siglec-8^FL^, supporting the hypothesis that membrane-proximal binding of Siglec-8 enhances ADCC. We then subjected EOL-1 and RS4;11 cell line pairs to in vitro cytotoxicity assays with Siglec-8^V-set^ CAR-modified KHYG-1 cells at various effector:target (E:T) cell ratios. Like what we found with Siglec-8^V-set^/CD3 BiAbs and Siglec-8 mAbs, these CAR-NK cells exerted greater cytotoxicity against AML and ALL cells expressing Siglec-8^V-set^ than cells expressing Siglec-8^FL^ (Figure 5E). In sum, these results demonstrated that moving the binding epitope closer to the cell membrane enhances the effector functions of Siglec-8 mAb-based therapies. This observation suggests the efficacy of Siglec-8-targeted therapies that engage T or NK cells as immune effector cells could be optimized by membrane-proximal targeting of Siglec-8 via one of the C2-set domains.

### 3.5. Generation of Siglec-8^C2-set^/CD3 BiAb- and NK Cell-Engaging Immunotherapies

**Figure 6:**
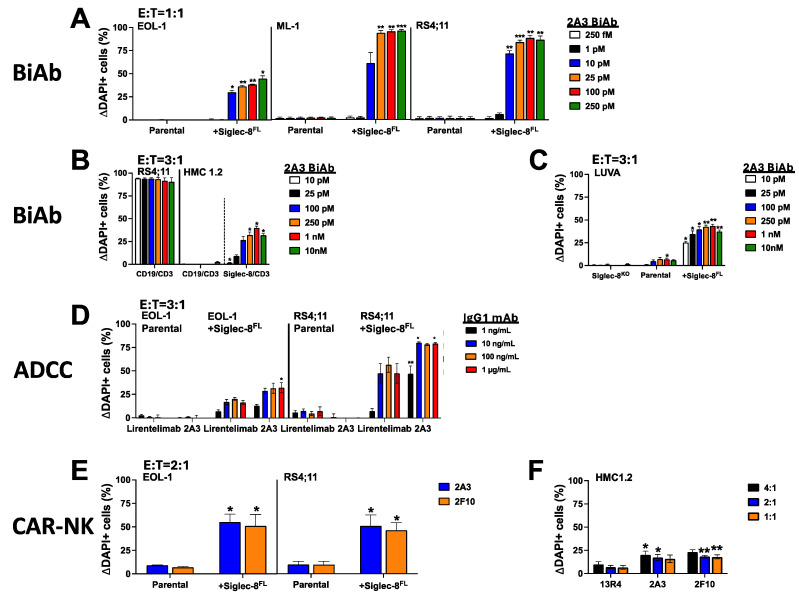
Cytolytic activity of Siglec-8^C2-set^ therapies engaging T or NK cells. (**A**) Parental EOL-1, ML-1, and RS4;11 cells and corresponding sublines overexpressing Siglec-8^FL^ were incubated with healthy donor T cells at an E:T cell ratio of 1:1 with increasing concentrations of a Siglec-8^C2-set^/CD3 BiAb (2A3). (**B**) Parental CD19+ RS4;11 cells and HMC-1.2 cells enriched for expression of endogenous Siglec-8 were incubated with healthy donor T cells at an E:T cell ratio of 3:1 with increasing concentrations of either a CD19/CD3 or a Siglec-8^C2-set^/CD3 BiAb (2A3). (**C**) Siglec-8 gene-edited LUVA cells (Siglec-8^KO^), parental LUVA cells, and LUVA cells overexpressing Siglec-8^FL^ were incubated with healthy donor T cells at an E:T cell ratio of 3:1 with increasing concentrations of a Siglec-8^C2-set^/CD3 BiAb (2A3). (**D**) Parental EOL-1 and RS4;11 cells and corresponding sublines overexpressing Siglec-8^FL^ were incubated with healthy donor NK cells at an E:T cell ratio of 3:1 with increasing concentrations of Siglec-8^C2-set^ mAb (2A3) or Siglec-8^V-set^ mAb (lirentelimab). (**E**) Cell lines as in (**D**) were incubated at an E:T cell ratio of 2:1 with KHYG-1 cells expressing a Siglec-8^C2-set^-directed CAR (2A3 or 2F10). (**F**) HMC-1.2 cells enriched for expression of endogenous Siglec-8 were incubated with KHYG-1 cells expressing either a Siglec-8^C2-set^-directed CAR (2A3 or 2F10) or a non-binding control CAR (13R4). For all experiments, non-viable target cells were enumerated after 48 h via flow cytometry. Change in dead cells with treatment compared to cells without treatment is shown (mean + SEM from 3 separate experiments). * *p* < 0.05; ** *p* < 0.01; *** *p* < 0.001.

Like lirentelimab, the majority of mAbs we isolated recognize the V-set domain of Siglec-8, indicating the immune-dominant epitopes are located on the outermost domain. Only three mAbs bound either the membrane-distal C2-set domain (2A3) or the membrane-proximal C2-set domain (2A7, 2F10), potentially serving as the basis for tool therapeutics that target Siglec-8 proximally. As one such therapeutic, we generated a Siglec-8^C2-set^/CD3 BiAb in the scFv-scFv format using binding sequences from 2A3 (a similar molecule based on 2F10 sequences failed to produce). As shown in Figure 6A–C, 2A3-derived Siglec-8^C2-set^/CD3 BiAbs were effective in eliminating EOL-1, ML-1, RS4;11, and LUVA cells that were transduced with Siglec-8^FL^ at very low concentrations. This BiAb was also effective in eliciting Siglec-8-specific cytotoxicity against Siglec-8-enriched HMC-1.2 cells and parental LUVA cells, both cell types that display Siglec-8 only at very low copy number. The observed activity of the CD19/CD3 BiAb against CD19+ RS4;11 cells but not CD19– HMC-1.2 cells strongly suggested that the cytotoxicity of the Siglec-8^C2-set^/CD3 BiAb against HMC-1.2 cells was not due to non-specific killing from T cells engaged via CD3 alone (Figure 6B). To test whether therapeutics binding Siglec-8 proximally could also elicit potent cytotoxicity when engaging NK rather than T cells, we first assessed ADCC effects. As an mAb (IgG1 format), 2A3 induced dose-dependent ADCC against RS4;11 and EOL-1 cells transduced with human Siglec-8^FL^ but not parental cell counterparts (Figure 6D), with greater cytotoxic effects than similar concentrations of lirentelimab (Figure 6D). No direct apoptotic effect was observed against leukemia cell lines transduced with Siglec-8^FL^ when Siglec-8 mAbs were incubated without NK cells. Moreover, KHYG-1 cells expressing Siglec-8^C2-set^-directed CARs generated with scFvs from 2A3 or 2F10 induced significant cytotoxicity against Siglec-8^FL^-transduced EOL-1 and RS4;11 cells (Figure 6E). These CAR KHYG-1 cells also exerted cytolytic effects against Siglec-8-enriched HMC-1.2 cells, albeit to a very modest extent (Figure 6F). Together, these studies demonstrated that Siglec-8^C2-set^-directed therapies engaging T or NK cells can be highly effective, even when target cells express limited amounts of Siglec-8.

### 3.6. Efficacy of Siglec-8^C2-set^-Directed Therapies against Primary Human Eosinophils

**Figure 7:**
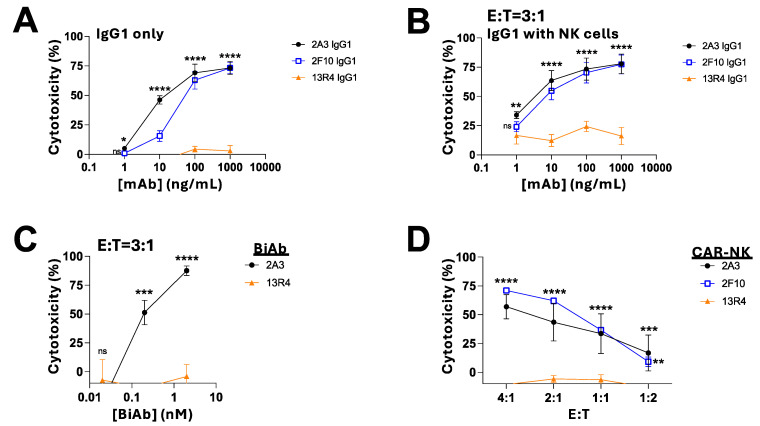
Efficacy of Siglec-8^C2-set^ therapies engaging T or NK cells against primary human eosinophils. (**A**) Eosinophils isolated from patients with eosinophilia were incubated with increasing concentrations of Siglec-8^C2-set^ mAb (2A3 or 2F10) or non-binding control mAb (13R4). (**B**) Same as (**A**) but with additional healthy donor NK cells present at an E:T cell ratio of 3:1. (**C**) Primary human eosinophils were incubated with healthy donor T cells at an E:T cell ratio of 3:1 with increasing concentrations of Siglec-8^C2-set^/CD3 BiAb (2A3) or a non-binding control BiAb (13R4). (**D**) Primary human eosinophils were incubated at various E:T cell ratios with KHYG-1 cells expressing either a Siglec-8^C2-set^-directed CAR (2A3 or 2F10) or a non-binding control CAR (13R4). For all experiments, live eosinophils were enumerated after 16–18 h via flow cytometry and change in live eosinophils with treatment compared to without treatment is shown (mean + SEM from 2 separate experiments using 2 different patient donors for eosinophils with at least 2 replicates/experiment). * *p* < 0.05; ** *p* < 0.01; *** *p* < 0.001; **** *p* < 0.0001; ns: not significant.

Like previously reported for lirentelimab [9], unconjugated Siglec-8^C2-set^ antibodies induced direct apoptosis in purified primary human eosinophils. Shown in Figure 7A, compared to a non-binding control mAb (13R4), Siglec-8^C2-set^ mAbs (2A3, 2F10; IgG1 format) induced dose-dependent apoptosis of eosinophils, even without prior activation of eosinophils with IL-5. With additional allogeneic primary human NK cells present, these Siglec-8^C2-set^ mAbs induced even greater cytotoxicity at low antibody concentrations (1–100 ng/mL), possibly because of ADCC (Figure 7B). Similarly, compared to a non-binding control BiAb (13R4), the Siglec-8^C2-set^/CD3 BiAb with sequences from 2A3 exerted dose-dependent T cell-mediated cytotoxicity against primary human eosinophils (Figure 7C). Finally, Siglec-8^C2-set^-directed CAR KHYG-1 cells built with 2A3 and 2F10 sequences exerted significantly greater, dose-dependent cytotoxicity against the primary human eosinophils compared to the non-binding control CAR-NK cells (13R4) (Figure 7D), demonstrating that Siglec-8^C2-set^-directed therapies engaging T or NK cells are highly potent against primary human eosinophils isolated from patients with eosinophilia.

## 4. Discussion

Siglecs are members of the immunoglobulin gene family implicated in promoting cell–cell interactions and regulating functions of cells in the innate and adaptive immune systems [28]. They are primarily found on hematopoietic and immune cells, mostly in a highly cell type-restricted pattern. This is true for Siglec-8, which is uniquely expressed on eosinophils, mast cells, and basophils (weakly) [1,29,30,31]. Displayed late during eosinophil and mast cell maturation, Siglec-8 is not found on hematopoietic stem cells [29]. This restricted expression pattern—together with the observation that unconjugated Siglec-8 mAbs cause caspase and/or reactive oxygen species-dependent apoptosis of eosinophils in vitro [32,33,34]—provides the rationale for Siglec-8-directed immunotherapies for eosinophilic and mast cell disorders. The benefit with lirentelimab in some patients [8,9,10,11] supports this notion, but more potent therapeutic modalities are likely necessary for other, more aggressive diseases involving eosinophils and mast cells.

Here, we raised new, fully human Siglec-8 mAbs as a basis for clinical therapeutics. Using several immunogens, we obtained a panel of mAbs with diversity regarding the epitopes to which they bind. Domain mapping studies indicated the majority of mAbs, like lirentelimab, recognize the V-set domain of Siglec-8, suggesting this outermost domain contains the immune-dominant epitopes on Siglec-8. This observation parallels that seen with Siglec-3 (CD33), where almost all mAbs recognize the V-set domain [15,18]. Nonetheless, a subset of the Siglec-8 mAbs we generated recognize epitopes on either the membrane-distal or membrane-proximal C2-set domain. This mAb diversity allows for tailoring the most ideal mAb to the desired treatment modality.

Previous studies demonstrated endocytic properties of Siglec-8 [27]. Consistent with endocytosis, all human mAbs we raised were internalized in a time- and Siglec-8-dependent fashion. While four of the five mAbs studied internalized with relatively similar kinetics, one mAb (2A3) internalized significantly more slowly, drawing attention to the possibility that some mAbs might be more suitable as carriers for toxic payloads (e.g., in the form of an antibody–drug conjugate or immunotoxin), whereas others might be better suited for applications for which limited internalization may be preferable (e.g., for targeted delivery of radioisotopes). Since 2A3 is the only mAb that binds the membrane-distal C2-set domain, we were unable to determine whether this difference in internalization kinetics between individual mAbs is due to specifics of the specific mAb clone or the domain it recognizes.

Because prior in vitro studies showed that unconjugated Siglec-8 mAbs inhibit mast cell function but cannot induce mast cell apoptosis [4,12,13,14], we focused our efforts on Siglec-8-directed therapies with T cell-engaging BiAbs and CAR-modified immune effector cells that likely have more potent effector functions. Our proof-of-principle experiments demonstrate that both Siglec-8/CD3 BiAbs and Siglec-8-directed NK CAR cells can be highly effective and highly specific against Siglec-8-expressing hematopoietic cells. Importantly, we found these therapeutics induced Siglec-8-specific cytotoxicity even against human mast cell lines with very minimal Siglec-8 expression, indicating such approaches could be effective even when target antigen expression is very low and other therapeutic modalities (e.g., radiolabeled or toxin-labeled mAbs) might fail. Together, these data provide a foundation for the development of Siglec-8/CD3 BiAbs and/or Siglec-8-directed CAR cells for eventual clinical application for patients with more aggressive diseases involving eosinophils and mast cells.

For several cell surface proteins, including two members of the Siglec family (CD22 and CD33), binding membrane-proximal epitopes was shown to enhance the efficacy of mAbs, CD3-directed BiAbs, or CAR T cells [15,16,35,36,37,38]. On the other hand, the relationship between epitope proximity and efficacy of mAb-based therapeutics has thus far not been studied for Siglec-8. To test this relationship, we utilized a strategy we and others have used before that is based on natural and artificial proteins where the targeted domain’s position relative to the cell membrane is varied. Specifically, we generated a construct where the V-set domain of Siglec-8 was positioned closer to the cell membrane, and used several Siglec-8^V-set^-directed therapeutics in comparative cytotoxicity experiments with cells expressing Siglec-8^FL^. Together, these studies—performed in cell line pairs that express well-matched levels of target antigens—demonstrate that membrane-proximal targeting of Siglec-8 enhances the cytolytic activities of unconjugated mAbs, Siglec-8/CD3 BiAbs, and Siglec-8-directed CAR-NK cells. While based on an artificial protein, this approach allows use of a single therapeutic for comparative assessments, thereby avoiding potential confounding data that could arise from mAbs with different affinities and/or the targeting of different binding epitopes. These data suggest one approach to optimize the efficacy of Siglec-8-targeted therapy is to build therapeutics from mAbs that bind to membrane-proximal regions of Siglec-8. To support this approach, we generated and tested several Siglec-8^C2-set^-directed tool therapeutics, demonstrating high anti-tumor activity in the context of unconjugated mAbs, T cell-directed BiAbs, and CAR-NK cells against not only human mast cell lines expressing Siglec-8 but also primary human eosinophils isolated from patients with eosinophilia. 

## 5. Conclusions

Taken together, having generated a series of diverse, human mAbs against Siglec-8, our preclinical data demonstrate Siglec-8-directed therapies can be highly potent, beyond what can be accomplished with unconjugated mAbs such as lirentelimab, supporting further development for use in patients with eosinophilic and mast cell disorders for whom current treatments are insufficiently effective.

## Data Availability

For original data and reagents, please contact the corresponding author (rwalter@fredhutch.org).

## Supplementary Materials

The following supporting information can be downloaded at: https://www.mdpi.com/article/10.3390/cancers16203476/s1, Figure S1: Parental EOL-1 and RS4;11 cells and corresponding sublines overexpressing similar levels of either Siglec-8^FL^ and Siglec-8^V-set^ were incubated with healthy donor NK cells at an E:T cell ratio of 3:1 with increasing concentrations of the Siglec-8^V-set^ antibody, 1H4, in IgG1 format; Table S1: Characteristics of patients with eosinophilia from whom primary human eosinophils were derived and used for in vitro experiments.
