# Supplementary material for: Optimizing Siglec-8-Directed Immunotherapy for Eosinophilic and Mast Cell Disorders
Source: Cancers (Basel). 2024 Oct 14;16(20):3476. doi: 10.3390/cancers16203476 (PMC11506601; doi:10.3390/cancers16203476)
Supplement: Supplementary file 1 [file cancers-16-03476-s001.zip › cancers-3233606-supplementary.pdf]

cancers

MDPI

Supplementary Materials

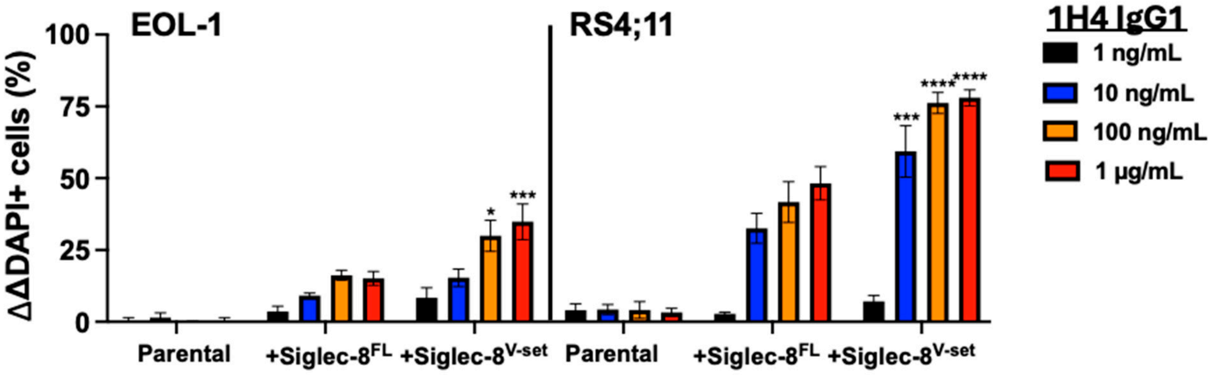

Figure S1. Parental EOL-1 and RS4;11 cells and corresponding sublines overexpressing similar levels of either Siglec-8^{FL} and Siglec-8$^{V\text{-}set}$ were incubated with healthy donor NK cells at an E:T cell ratio of 3:1 with increasing concentrations of the Siglec-8$^{V\text{-}set}$ antibody, 1H4, in IgG1 format. Non-viable target cells were enumerated after 48 hours via flow cytometry. Mean+SEM values from at least 3 separate experiments are shown. *P<0.05; ***P<0.001; ****P<0.0001.

Table S1. Characteristics of patients with eosinophilia from whom primary human eosinophils were derived and used for in vitro experiments.

Patient	Eosinophil count (x10^9/L)	Eosinophil Percentage (%)	White blood cell count (x10^9/L)	Diagnosis
1	13.41	69	19.43	Angioimmunoblastic T-cell lymphoma
2	2.09	28	7.48	Acute myeloid leukemia (trisomy 8)
